# “Two Birds with One Stone”: F Doping Ni–Co Hydroxide as High-Performance Cathode Material for Aqueous Zn Batteries

**DOI:** 10.3390/nano12101780

**Published:** 2022-05-23

**Authors:** Wen Liu, Qiwen Zhao, Yunyun Wang, Yuejiao Chen, Libao Chen

**Affiliations:** State Key Laboratory of Powder Metallurgy, Central South University, Changsha 410083, China; levinesky12@163.com (W.L.); qiwen9259@163.com (Q.Z.); cloudsays@163.com (Y.W.)

**Keywords:** F doping, Ni–Co hydroxide, aqueous Zn battery, hierarchical morphology, tunable composition

## Abstract

Cathode materials have impeded the development of aqueous Zn batteries (AZBs) for a long time due to their low capacity and poor cycling stability. Here, a “two birds with one stone” strategy is devised to optimize the Ni–Co hydroxide cathode material (NCH) for AZBs, which plays an essential role in both composition adjustment and morphology majorization. The F-doped Ni–Co hydroxide (FNCH) exhibits a unique nanoarray structure consisting of the 2D flake-like unit, furnishing abundant active sites for the redox reaction. A series of analyses prove that FNCH delivers improved electrical conductivity and enhanced electrochemical activity. Contributing to the unique morphology and adjusted characteristics, FNCH presents a higher discharge-specific capacity, more advantageous rate capability and competitive cycling stability than NCH. As a result, an aqueous Zn battery assembled with a FNCH cathode and Zn anode exhibits a high capacity of 0.23 mAh cm^−2^ at 1 mA cm^−2^, and retains 0.10 mAh cm^−2^ at 10 mA cm^−2^. More importantly, the FNCH–Zn battery demonstrates no capacity decay after 3000 cycles with a conspicuous capacity of 0.15 mAh cm^−2^ at 8 mA cm^−2^, indicating a superior cycling performance. This work provides a facile approach to develop high-performance cathodes for aqueous Zn batteries.

## 1. Introduction

With the advantages of high energy density and admirable cycling stability, Li ion batteries (LIBs) occupy a dominant position in the fields of portable electronics and electrical vehicles [1]. Unfortunately, cost and safety issues still hinder the further development of LIBs [2,3,4]. The usage of organic electrolytes not only improves the potential safety hazard but also increases the cost of battery packaging [5,6]. Alternatively, aqueous zinc batteries (AZBs) exhibit huge competitiveness in the field of wearable portable electronics due to their high safety and low cost [7]. Aqueous electrolytes possess higher security and ion conductivity compared to flammable and toxic organic electrolytes, thus providing an intrinsic safety guarantee and excellent rate capability for batteries [8]. Furthermore, benefiting from the balanced characteristic of Zn metal in aqueous electrolytes, AZBs perform a high theoretical capacity and striking cost-effectiveness, stimulating vast research interests around the world [9,10,11].

Regardless of the considerable advancements in AZBs, the unsatisfactory energy density and inferior cycling stability are still two obstacles that limit the applications of AZBs [12]. The Zn metal anode faces a series of challenges, like dendrite formation, corrosion and passivation, which shorten the working lifespan of the batteries [2]. With regard to cathode materials, two pivotal factors seriously affect the performance of the AZBs. On the one hand, the cathode materials show an unmatched capacity compared to the Zn anode, resulting in a disappointing energy density [13]. On the other hand, cathode materials are always plagued by the thorny troubles of deformation and dissolution during repeated charge/discharge cycles, leading to frustrating cycling stability [14,15]. Consequently, it is urgently desirable to develop cathode materials with high capacity and superior cycling stability for AZBs with exceptional performance.

Recently, a great deal of efforts has been conducted to improve the performance of cathode materials for AZBs. For instance, Qiu and coworkers reported electron-density-modulated NiCo_2_O_4_ nanosheets as the cathode and, as a result, the incorporation of nitrogen regulates the electronic and electrochemical properties of the NiCo_2_O_4_ nanosheets [16]. Furthermore, heteroatom doping is an effective approach to regulate the material composition for better electrical conductivity and electrochemical activity [17,18,19]. Qiao and coworkers reported NiCo–OH nanothorns coated CuO nanowire arrays as cathode materials; the 3D construction allows an effective exposure of the active material in electrolytes and provides more reaction sites to promote electrochemical reactions on the cathode [20]. Xie and coworkers demonstrated hierarchical NiCo_2_O_4_@CoMoO_4_@Co_3_O_4_ arrayed structures for AZBs, and such an electrode design possesses abundant electrochemical active sites and facile ion diffusion towards high performance [21]. By properly optimizing the morphology, the overall electrochemical kinetics will be improved, leading to better electrochemical performance.

Taking all factors into consideration, we propose an F-doping strategy to ameliorate the Ni–Co hydroxide cathode material (FNCH) for AZBs. The F-doping strategy not only adjusts the composition, but also optimizes the morphology of the cathode material. The electronic structure of the material has changed and the electrochemical activity is enhanced commendably with the introduction of F doping. The FNCH demonstrates an improved electrical conductivity and a lower OH^−^ adsorption energy that suggests superior electrochemical performance compared to undoped counterparts. Notably, the as-doped FNCH provides a higher specific capacity of 0.41 mAh cm^−2^ at 1 mA cm^−2^, and a sterling rate capability of 57.8% as current density increased to 15 mA cm^−2^. Moreover, a more competitive capacity of 0.31 mAh cm^−2^ after 2500 cycles is achieved by FNCH compared to NCH (0.05 mAh cm^−2^) at 10 mA cm^−2^. Furthermore, assembled with a Zn anode, an FNCH–Zn battery presents extraordinary superior electrochemical performance compared to a NCH–Zn battery, indicating that the cathode material with high performance was prepared successfully by an F-doping strategy.

## 2. Experimental

### 2.1. Materials Synthesis

All chemicals were analytical grade and used without any further purification. The F-doping strategy was achieved by a facile hydrothermal method. Firstly, the Ni foam was cleaned in dilute HCl, deionized water and absolute ethanol by ultrasonication and dried thoroughly. A total of 0.291 g Ni(NO_3_)_2_·6H_2_O, 0.582 g Co(NO_3_)2·6H_2_O, 0.9 g urea and 0.111 g ammonium fluoride (NH_4_F) were dissolved in 60 mL purified water. After magnetic stirring, the mixed solution was transferred into a 100 mL autoclave where a piece of pretreated Ni foam (2 × 4 cm^2^) was soaked for 20 min. Then the autoclave was sealed and heated at 120 °C for 5 h. After the hydrothermal reaction, the Ni Foam with prepared materials was taken out and washed several times with deionized water. Then, the samples were dried and named as FNCH. For comparative study, the undoped material (NCH) was also prepared without fluorine under the same condition. The mass loading of FNCH and NCH was calculated to be 2.14 mg cm^−2^ and 2.02 mg cm^−2^, respectively.

### 2.2. Material Characterization

The phase structure of samples was characterized by X-ray diffraction (XRD, Rigaku D/Max-2550 VB, Cu Kα radiation, λ = 1.5406 Å). The surface elements of the samples were studied using X-ray photoelectron spectroscopy (XPS, Thermo Fisher Scientific Escalab 250, New York, NY, USA, Al Kα 1486.6 eV). The morphology was observed by scanning electron microscopy (SEM, TESCAN MIRA3 LMH, Brno, Czech). The details of morphology and crystal structure were studied by a transmission electron microscope (TEM, Tecnai G2 F20, FEI, Hillsborough, OR, USA).

### 2.3. Electrochemical Measurements

The electrochemical measurements were conducted with an electrochemical workstation (IVIUM) at room temperature. The sample was cut into 1 × 2 cm^2^ for electrochemical performance tests. The electrochemical performances of the individual electrode were measured in a three-electrode system, with a platinum foil as the counter electrode and Hg/HgO as the reference electrode, in 3 M KOH aqueous solution. The cyclic voltammetry (CV) curves were measured in the potential region from −0.2 to 0.6 V. The voltage window of galvanostatic charge–discharge (GCD) was 0–0.52 V, and the EIS range was 100 kHz to 0.01 Hz with the amplitude of 5 mV. The Zn batteries were assembled using FNCH and NCH as a cathode, Zn plate as the anode with 3 M KOH as the electrolyte. The voltage window of the CV and GCD test were 1–2 V and 1.4–1.9 V, respectively.

### 2.4. Density Function Theory Calculations

First-principle calculations were performed by the density functional theory (DFT) using the Vienna Ab-initio Simulation Package (VASP) package. The generalized gradient approximation (GGA) with the Perdew−Burke−Ernzerhof (PBE) functional were used to describe the electronic exchange and correlation effects. Uniform G-centered k-point meshes with a resolution of 2π × 0.03 Å^−1^ and Methfessel-Paxton electronic smearing were adopted for the integration in the Brillouin zone for geometric optimization. The simulation was run with a cutoff energy of 500 eV throughout the computations. These settings ensure convergence of the total energies to within 1 meV per atom. Structure relaxation proceeded until all forces on the atoms were less than 1 meV Å^−1^ and the total stress tensor was within 0.01 GPa of the target value. Due to the strong correlation of d electrons in Ni and Co, a U−J parameter of 6.45 and 4.90 eV was applied.

For the NCH and FNCH systems, the reaction surfaces were (011) planes. For each system, the bottom four layers of atoms were fixed and the vacuum space was 15 Å. The adsorption free energy for adsorbed OH^−^ species was calculated by the following equation:

(1) $$\Delta \mathrm{Gads}=E\left(\mathrm{total}\right)-E\left(\mathrm{surface}\right)-E\left({\mathrm{OH}}^{-}\right)$$

where E(total), E(surface) and E(OH^−^) are the energies of the surface absorbed OH^−^ species, the selected surface and OH^−^ species, respectively.

## 3. Results and Discussion

**Figure 1:**
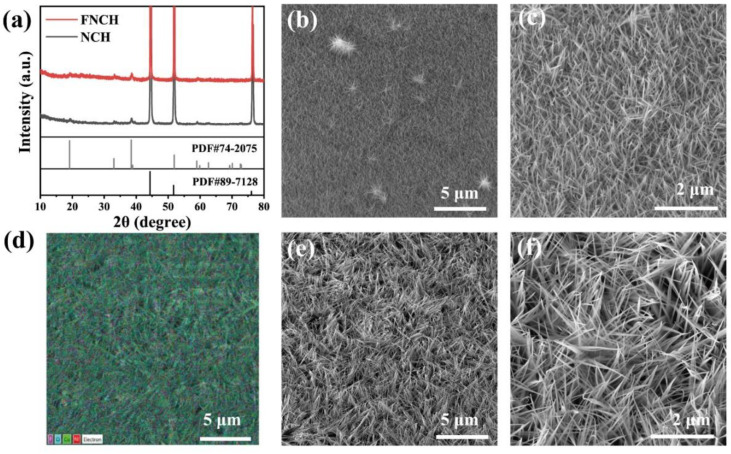
Characterization of phase and morphology. (**a**) XRD pattern of FNCH and NCH; (**b**,**c**) SEM images of NCH; (**d**) SEM EDX mapping of FNCH; (**e**,**f**) SEM images of FNCH.

The F-doped Ni–Co hydroxide (FNCH) was grown on the conductive Ni foam substrate through a simple hydrothermal method. Ni foam not only provides a quick electron transfer process but also affords a large surface area for higher mass loading. The XRD patterns of both two samples display three high-intensity peaks (Figure 1a), which are the characteristic peaks of nickel metal (JCPDS no.89-7128). Apart from Ni foam, the peaks at 19.2°, 33.1° and 38.5° can be observed in the XRD patterns of both FNCH and NCH, which are well indexed to the (001), (100) and (011) planes of nickel hydroxide (JCPDS no.74-2075). F doping shows negligible influence in crystal structure, without an obvious change in the diffraction peak position in the XRD pattern (Figure 1a). We propose the reason should be the fact that the ionic radius of F^−^ (0.133 nm) is quite similar to that of O^2−^ (0.140 nm) [22,23]. Indeed, when the XRD pattern is locally enlarged between 30°and 40° (Figure S2), the peaks of the fluorine-doping sample shift slightly to a larger 2θ value, reflecting the shrinkage of the crystal lattice. The shrinkage of the lattice may be caused by the substitution of the O^2−^ lattice by F^−^ which proves fluorine doping [24]. Specifically, the peak intensity of FNCH is generally stronger than that of NCH, indicating the higher mass loading of the doped sample. Scanning electron microscopy (SEM) is used to investigate the morphology and structure of prepared materials. Both FNCH and NCH cover the smooth surface of Ni foam (Figure S1) uniformly after hydrothermal synthesis, but the FNCH exhibits distinctly different morphology. As shown in Figure 1b,c, NCH shows a simple structure of pure nanowires. Figure 1d shows the SEM element distribution of FNCH; F element distributes evenly on the nanoarray, as well as Ni, Co and O elements. As revealed in Table S1, the atomic percentages of fluorine, nickel, cobalt and oxygen are 4.7:14:19:62.3. From the high magnification SEM images of FNCH (Figure 1e,f), a unique nanoarray grown vertically on the surface and the array unit shows a hierarchical structure, which consists of a flake-like body and extended nanowires.

**Figure 2:**
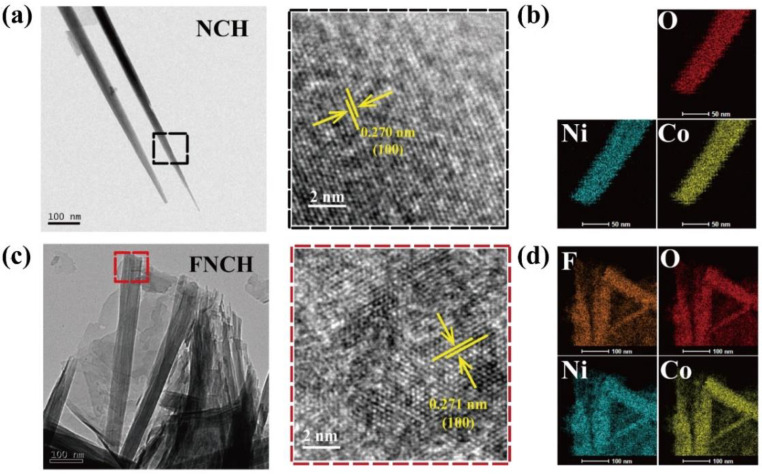
TEM images of NCH (**a**) and FNCH (**c**); TEM EDX mapping of NCH (**b**) and FNCH (**d**).

Transmission electron microscopy (TEM) is applied to further study the 1D nanowire structure of NCH and the unique structure of FNCH. The array unit of NCH and FNCH are detached from the Ni foam by ultrasonication, and their TEM and HRTEM images are shown in Figure 2a,c, respectively. Single nanowire with a diameter of 20~30 nm can be observed in NCH and the 0.270 nm lattice fringe is indexed to the (100) plane of nickel hydroxide (Figure 2a), consistent with the results of XRD and SEM. FNCH displays a similar interplanar distance of 0.271 nm with NCH, corresponding well to the XRD result. The array unit of FNCH exhibits a hierarchical structure where the nanowires staggered to form a 2D flake-like unit. Combining the TEM and SEM images, FNCH demonstrates a unique structure of nanoarray with a vertically aligned hierarchical unit on Ni foam, which may shorten the diffusion path of the electrolyte ion. The TEM energy dispersive X-ray spectroscopy (EDX) mapping result reveals that NCH and FNCH have an even element distribution on their array unit, but the F element only appears in FNCH while Ni, Co and O elements exist in both NCH and FNCH, suggesting the uniform doping of the F element (Figure 2b,d).

**Figure 3:**
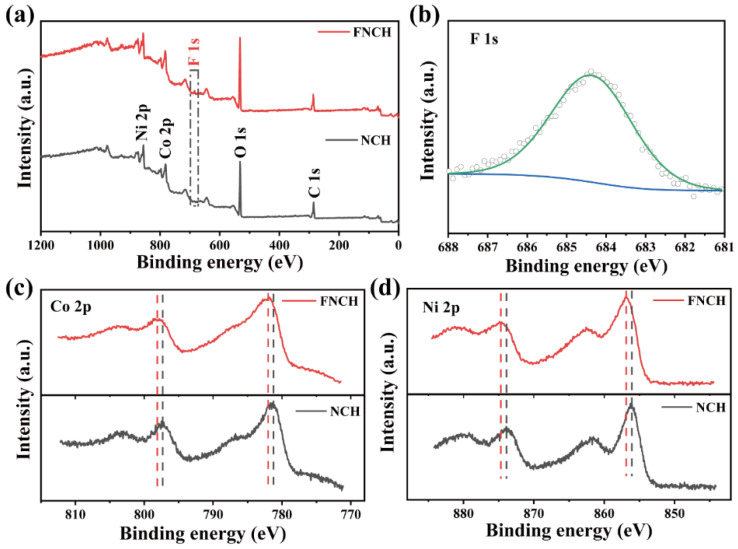
Analysis of the state of surface. (**a**) XPS survey spectra of FNCH and NCH; (**b**) F 1s spectra of FNCH; Comparison of Co 2p (**c**) and Ni 2p spectra (**d**) between FNCH and NCH.

X-ray photoelectron spectroscopy (XPS) measurement is conducted to acknowledge the chemical states of surface elements. From the XPS survey spectra, the F element can be only observed in FNCH, while the characteristic peaks of Ni, Co and O exist in both FNCH and NCH. Figure 3b shows the high-resolution spectra of F 1s, the peak located at 684.31 eV is attributed to the F–metal bonding, which confirms the doping of the F element [25]. Figure 3c,d compared the high-resolution spectra between FNCH and NCH. Both Ni 2p and Co 2p peaks in the spectra of FNCH shift slightly towards a higher binding energy compared to NCH, due to the incorporation of the F element [26]. The high electronegativity provides the F element with a strong electron-withdrawing ability, and thus improves the electron binding energy [27,28]. Obviously, F doping is achieved successfully through a facile hydrothermal synthesis and plays a momentous role in composition adjustment and morphology control.

**Figure 4:**
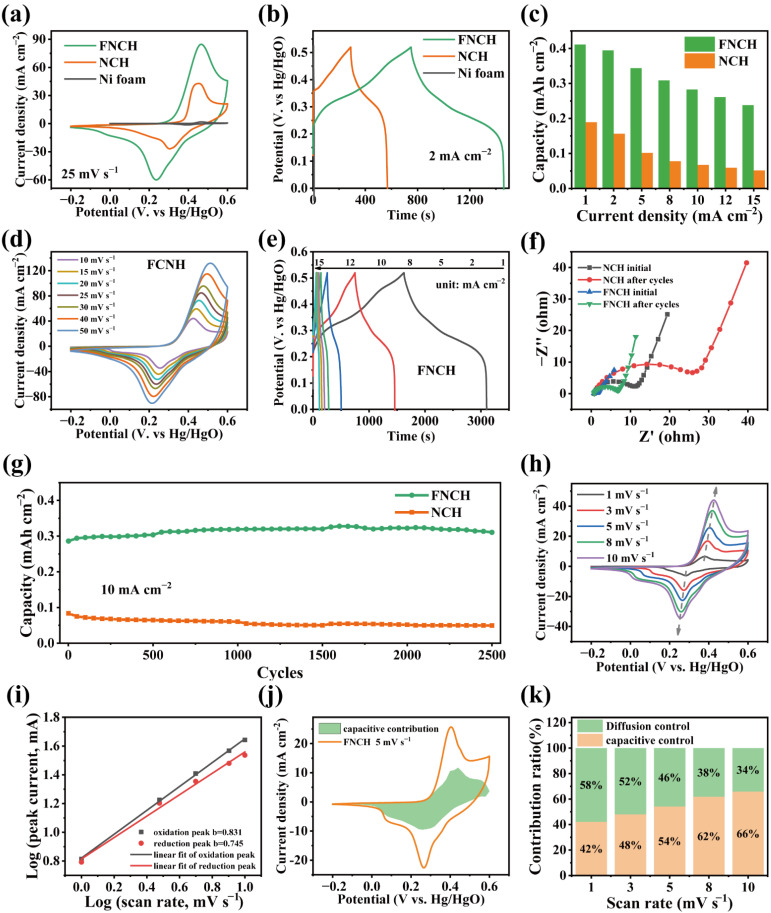
Electrochemical performance tests of FNCH and NCH materials. CV curves (**a**) and GCD curves (**b**) comparisons of FNCH and NCH; (**c**) capacity at various current of FNCH and NCH; (**d**) CV curves and (**e**) GCD curves of FNCH under different conditions; (**f**) Nyquist plot of FNCH and NCH before and after cycles. (**g**) Cycling performance of FNCH and NCH; (**h**) CV curves of FNCH for b value fitting; (**i**) b value fitting result; (**j**) CV curves of origin data and fitting data at 5 mV s^−1^. (**k**) Contribution ratio at different scan rates.

To investigate the F-doping effect on the electrochemical performance of the material, relevant electrochemical tests are conducted using a three-electrode system in 3M KOH electrolyte. Figure 4a compares the CV curves of FNCH and NCH at 25 mV s^−1^ from −0.2 to 0.6 V, both FNCH and NCH show a typical butterfly-like shape with a pair of redox peaks, and it was found that the redox peaks are at 0.464 V and 0.237 V for FNCH, and the redox peaks are at 0.452 V and 0.306 V for NCH, corresponding to the proton insertion/extraction reaction [29]. Simply, the material reacts with the OH^−^ during the charge and discharge process, accompanied by the chemical valence change of Ni and Co species and the electron transfer [30,31,32]. Obviously, FNCH elaborates a couple of border redox peaks and a larger CV area, manifesting a better electrochemical performance. In addition, when scan rates improve from 10 to 50 mV s^−1^, the anodic and cathodic peak current increases and the peak position shifts to positive and negative potentials, leading to the increased CV areas (Figure 4d and Figure S3a), yet, FNCH always shows a larger CV area.

Figure 4b expounds the GCD curves of FNCH and NCH at the current density of 2 mA cm^−2^ within the potential window from 0 to 0.52 V. Both curves are nonlinear and present a clear platform during the charge and discharge process, which agree well with the CV test results, indicating the battery-like characteristic. The curve of FNCH shows a longer discharge time and larger discharge area, suggesting a better electrochemical performance. As shown in Figure 4c, the capacity of two samples at the various current density is calculated from the GCD curves (Figure 4e and Figure S3b) and plotted as a bar graph. FNCH delivers a higher capacity than NCH when discharged at the same current density, revealing that the FNCH electrode with 2D hierarchical structure is superior to NCH. When current density improves from 1 mA cm^−2^ to 15 mA cm^−2^, FNCH exhibits a higher rate capability of 58% than NCH (27%), suggesting the quicker electron transfer and electrolyte ion diffusion of FNCH.

The cycling stability is measured through a long-term GCD test at the current density of 10 mA cm^−2^. Figure 4g shows the comparison of the cycling stability between NCH and FNCH; FNCH still provides a higher capacity of 0.31 mAh cm^−2^ after 2500 cycles than NCH (0.05 mAh cm^−2^). In the meantime, the capacity of FNCH is increasing along with the GCD cycling, leading to the capacity retention of 109%. As for NCH, the capacity fades gradually within the 3000 cycles and the capacity retention is as low as 59%. Evidently, FNCH presents a more remarkable cycling stability than NCH, which is attributed to the 2D hierarchical structure that provides enough space for volume expansion and stress release during the charge and discharge process. EIS measurements are used to investigate the electrochemical kinetics, and the Nyquist plots are showed in Figure 4f. All of the samples account a similar value of R_es_ (the intersection between the curves and the real axis) due to the usage of the Ni foam substrate [33]. Differently, NCH displays an obvious semicircle at high frequency, while FNCH exhibits a small semicircle that represents a lower charge transfer resistance (R_ct_). The charge transfer resistance values of FNCH and NCH can be fitted to be 0.9 Ω and 8.9 Ω, respectively. The EIS results suggest that FNCH possesses a quick ion diffusion rate and low charge transfer resistance, which is instrumental in a better electrochemical performance compared to NCH. After a long-term cycling test, both FNCH and NCH show an increased R_ct_ due to the volume expansion and degradation of active material during a repeated electrochemical reaction [34]. However, FNCH exhibits a lower R_ct_ value of 5.4 Ω than NCH (22.3 Ω) after cycling, suggesting a more stable cycling performance of FNCH.

The electrochemical energy storage behavior of FNCH is further researched using the CV test. As shown in Figure 4h, the anodic and the cathodic peak currents increase when scan rate is improved. The relationship between peak current and scan rate can be analyzed by applying the power law equation: $i=a\nu^{b}$, where the parameter b value represents the electrochemical characteristic of materials [35]. The b value of 0.5 indicates the diffusion-control process and the b value of 1 suggests the capacitive-control process. According to the fitted b value in Figure 4i, anodic and cathodic peaks display a b value of 0.871 and 0.745 (between 0.5 and 1), respectively, implying the energy storage including the contributions of diffusion-control and capacitive-control [36]. Additionally, the contribution ratio can be divided using the equation: $i\left(V\right)=k_{1}v+k_{2}v^{\frac{1}{2}}$, the $k_{1}v$ and the $k_{2}v^{\frac{1}{2}}$ represent the capacitive-control contribution and diffusion-control contribution [37]. The fitted $k_{1}v$ curve and the original CV curve at 5 mV s^−1^ are described in Figure 4k, and the capacitive contribution ratio of 42% is obtained from the area ratio of two curves. Figure 4j shows the contribution ratio at different scan rates, the capacitive-control contribution ratio increases from 56% to 66% when the scan rate is improved from 1 mV s^−1^ to 10 mV s^−1^, hinting fast electrochemical reaction kinetics of FNCH.

**Figure 5:**
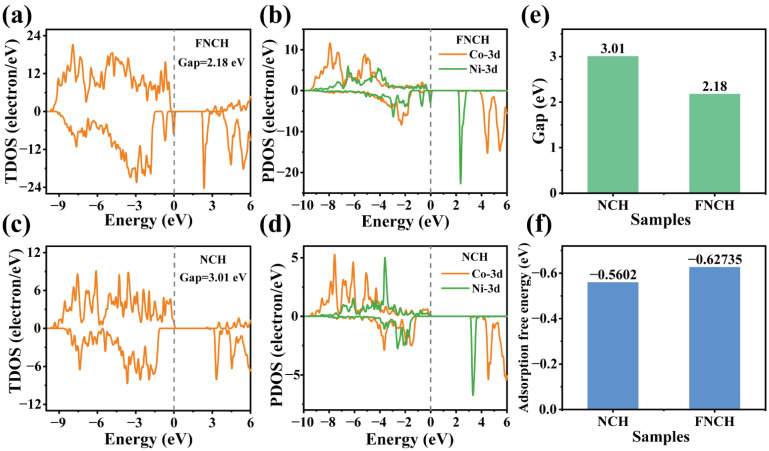
TDOS of FNCH (**a**) and NCH (**c**); PDOS of Ni-3d and Co-3d of FNCH (**b**) and NCH (**d**); (**e**) gap values of FNCH and NCH; (**f**) OH^−^ adsorption energy of FNCH and NCH.

Density functional theory (DFT) computation is conducted to reveal the F-doping effect on the intrinsic characteristic of the material (models of the structure are shown in Figures S4 and S5). It can be easily concluded from the calculation result that both NCH and FNCH exhibit a clear band gap, corresponding to the semiconductor characteristic (Figure 5a–d). Owing to the hybridization of the 2p orbit of F^−^, the denser electrons are collected in a valence band after the introduction of fluorine, which is beneficial for narrowing band gap and improving electrical conductivity in FNCH [38]. As expected, FNCH shows a lower band gap of 2.18 eV than NCH (3.01 eV), suggesting improved electrical conductivity of FNCH after F doping. As mentioned before, the energy storage is dominated by proton insertion/extraction and OH^−^ plays a vital role in the redox reaction [39,40]. Therefore, the OH^−^ adsorption energy of FNCH and NCH are calculated and the corresponding results are displayed in Figure 5f. The adsorption energy of FNCH and NCH is −0.63 eV and −0.56 eV, respectively. The lower adsorption energy of FNCH means enhanced electrochemical activity, which is beneficial for the proton insertion/extraction reaction. The results of theory calculation reveal that F doping can effectively adjust the electronic structure and improve the OH^−^ adsorption ability for high-performance cathode materials.

**Figure 6:**
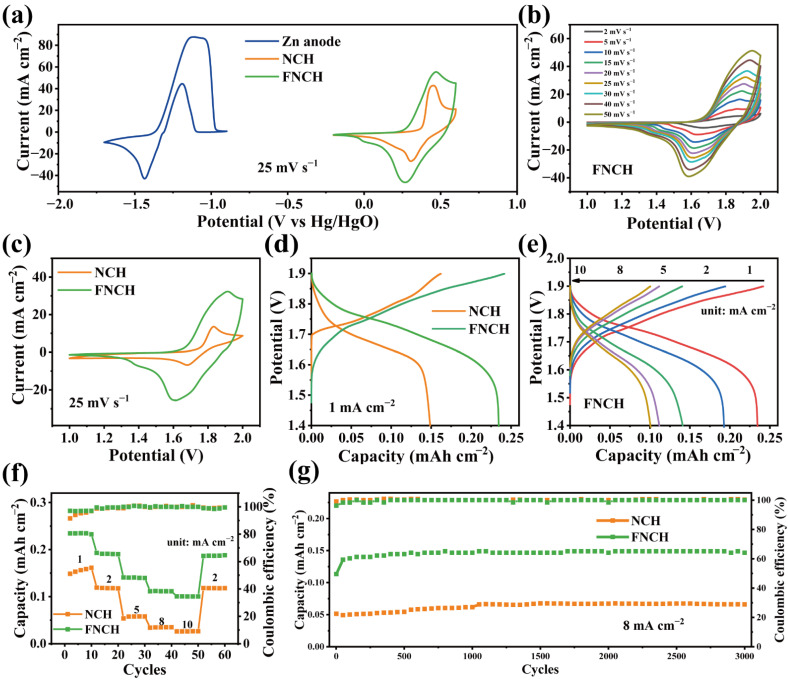
Electrochemical performance tests of FNCH–Zn and NCH–Zn batteries. (**a**) CV curves of Zn anode, FNCH and NCH cathode material at 25 mV s^−1^. (**b**) CV curves of FNCH–Zn battery. (**c**) CV curve comparison of FNCH–Zn and NCH–Zn battery. (**d**) GCD curves of FNCH–Zn battery. Comparison of GCD curve (**e**), rate capacity (**f**) and cycling performance (**g**) for FNCH–Zn and NCH–Zn battery.

Based on the above study, aqueous Zn batteries are assembled using NCH or FNCH as the cathode electrode and Zn plate as the anode electrode with a 3M KOH electrolyte in a two-electrode system. The CV curve of the Zn anode shows a typical battery-like shape as well as FNCH and NCH (Figure 6a) and the low redox potential can provide a high working voltage for the Zn battery to achieve a high energy density [41,42]. The CV curves of the FNCH–Zn and NCH-Zn battery at various scan rates are shown in Figure 6b and Figure S6a, and the battery presents a similar shape without obvious distortion when scan rate increases from 2 to 50 mV s^−1^, suggesting low polarization and fast charge transfer. The CV curve comparison of the FNCH–Zn battery and NCH–Zn battery at 25 mV s^−1^ are illustrated in Figure 6c; the FNCH–Zn battery exhibits a larger CV area than the NCH–Zn battery, indicating a better electrochemical performance. As revealed in Figure 6d, the FNCH–Zn battery delivers a capacity of 0.23 mAh cm^−2^ at 1 mA cm^−2^, which is much higher than the NCH–Zn battery (0.15 mAh cm^−2^). As the current density increased to 10 mA cm^−2^, the FNCH–Zn battery still maintains a high capacity of 0.1 mAh cm^−2^, while the capacity of the NCH–Zn battery is below 0.05 mAh cm^−2^ (Figure 6e and Figure S6b). Both the FNCH–Zn and NCH–Zn batteries exhibit a good rate capability when discharged at various current densities, but the capacity of the FNCH–Zn battery is always higher (Figure 6f). From the cycling performance of the batteries in Figure 6g, the FNCH–Zn battery delivers a higher capacity than the NCH battery without any decay after 3000 cycles, revealing exceedingly excellent cycling stability. Consequently, FNCH shows better electrochemical performance when applied in aqueous Zn batteries. With the high capacity and outstanding cycling stability, the modified FNCH material is proved to be a potential cathode for high performance AZBs.

## 4. Conclusions

In summary, a facile approach of F doping has been proposed to modify Ni–Co hydroxide cathode material (NCH) on composition and morphology. The as-doped FNCH material provides a specific capacity of 0.41 mAh cm^−2^ at 1 mA cm^−2^, a high-rate capability of 58% at 15 mA cm^−2^, and a capacity of 109% after 2500 cycles at 10 mA cm^−2^. The effect of F doping is investigated by combining electrochemical tests with analysis. FNCH exhibits a 2D hierarchical nanoarray structure with abundant active sites and fast electrolyte ion diffusion. As a result, FNCH exhibits a higher capacity, better rate capacity and more stable cycling performance than NCH. Finally, the aqueous Zn batteries are constructed using FNCH/NCH as a cathode and the FNCH–Zn battery presents a better electrochemical performance than the NCH–Zn battery, further confirming the effectiveness of an F-doping strategy. This work provides a facile but effective approach to develop high-performance cathode material for aqueous Zn batteries. Combining composition adjustment with morphology control, this feature affords the “kill two birds with one stone” strategy.

## Data Availability

Not applicable.

## Supplementary Materials

The following supporting information can be downloaded at: https://www.mdpi.com/article/10.3390/nano12101780/s1, Figure S1: SEM image of Ni foam substrate; Figure S2: Comparison of diffraction peaks between 32°to 40°in FNCH and NCH; Figure S3: CV curves (a) and GCD curves (b) of NCH; Figure S4: Ball and stick models of the structure of NCH (a) and FCNH (b); Figure S5: Models of the calculation of OH- adsorption on NCH (a) and FNCH (b); Figure S6: CV curves (a) and GCD curves (b) of NCH//Zn battery; Table S1: The results of SEM EDX mapping.
