# Supplementary material for: “Two Birds with One Stone”: F Doping Ni–Co Hydroxide as High-Performance Cathode Material for Aqueous Zn Batteries
Source: Nanomaterials (Basel). 2022 May 23;12(10):1780. doi: 10.3390/nano12101780 (PMC9144373; doi:10.3390/nano12101780)
Supplement: Supplementary file 1 [file nanomaterials-12-01780-s001.zip › nanomaterials-1716246-supplementary.pdf]

nanomaterials

Supporting Information

"Two Birds with One Stone": F Doping Ni–Co Hydroxide as High-Performance Cathode Material for Aqueous Zn Batteries

Wen Liu, Qiwen Zhao, Yunyun Wang, Yuejiao Chen * and Libao Chen *

State Key Laboratory of Powder Metallurgy, Central South University, Changsha 410083, China; levinesky12@163.com (W.L.); qiwen9259@163.com (Q.Z.); cloudsays@163.com (Y.W.)
* Correspondence: cyj.strive@csu.edu.cn (Y.C.); lbchen@csu.edu.cn (L.C.)

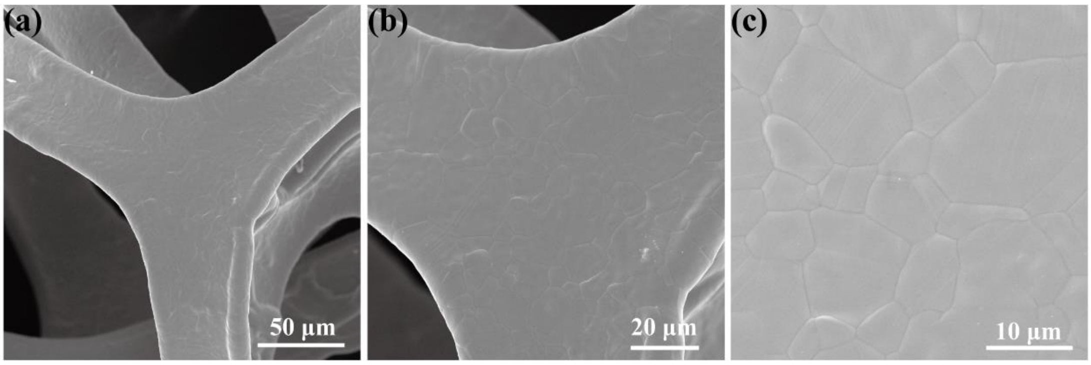

Figure S1. SEM image of Ni foam substrate (**a**) 50 μm; (**b**) 20 μm; (**c**) 10 μm.

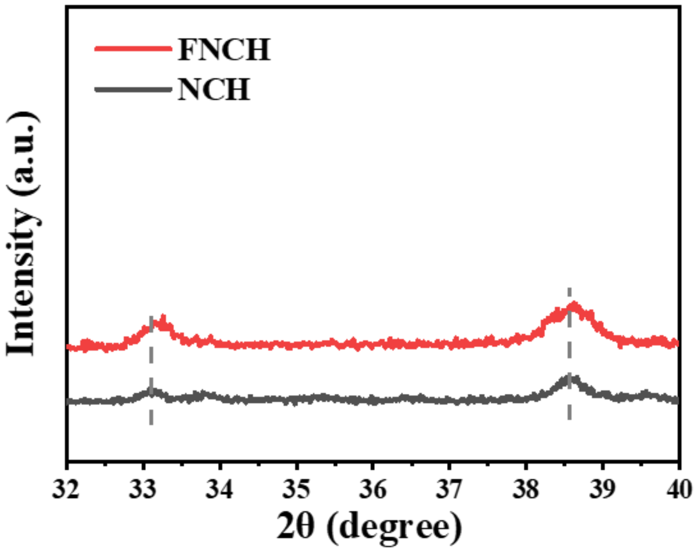

Figure S2. Comparison of diffraction peaks between 32°to 40°in FNCH and NCH.

Table S1. The results of SEM EDX mapping.

Element	NCH (Atomic%)	FNCH (Atomic%)
O	64.1	62.3
Co	20.8	19
Ni	14.7	14
F	0.4	4.7

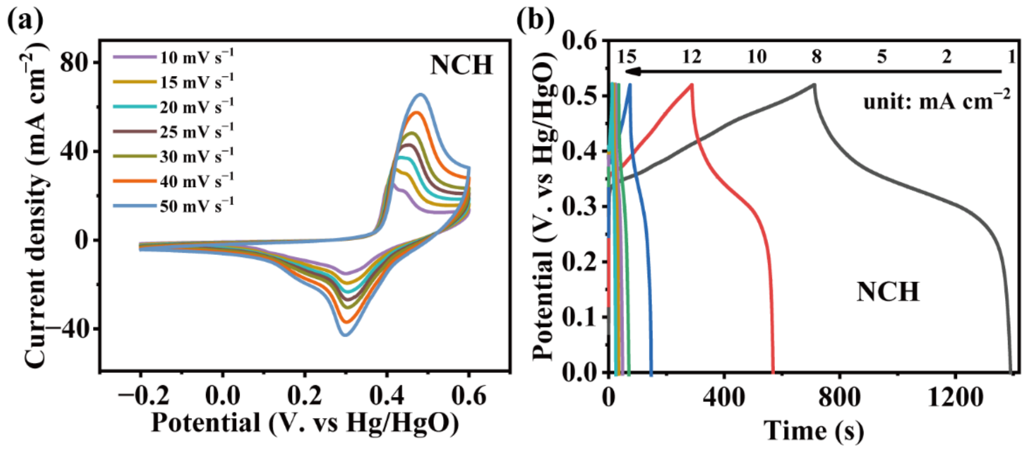

Figure S3. CV curves (**a**) and GCD curves (**b**) of NCH.

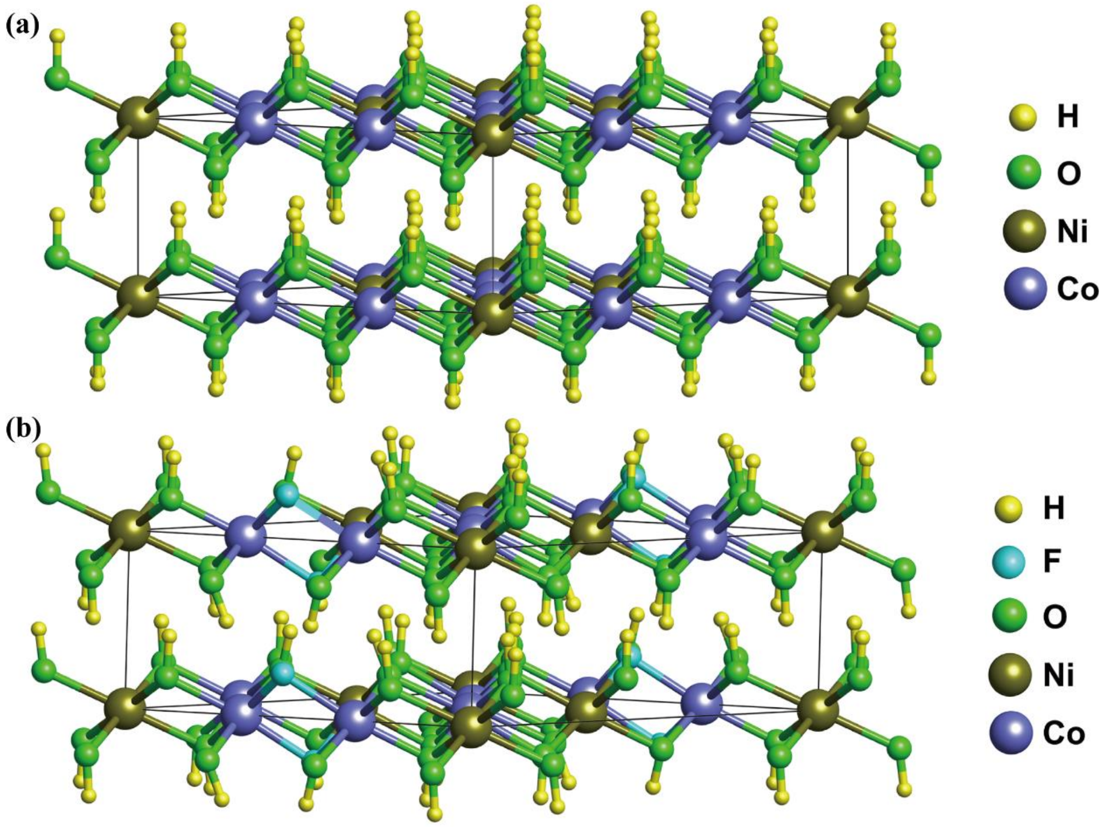

Figure S4. Ball and stick models of the structure of NCH (**a**) and FCNH (**b**).

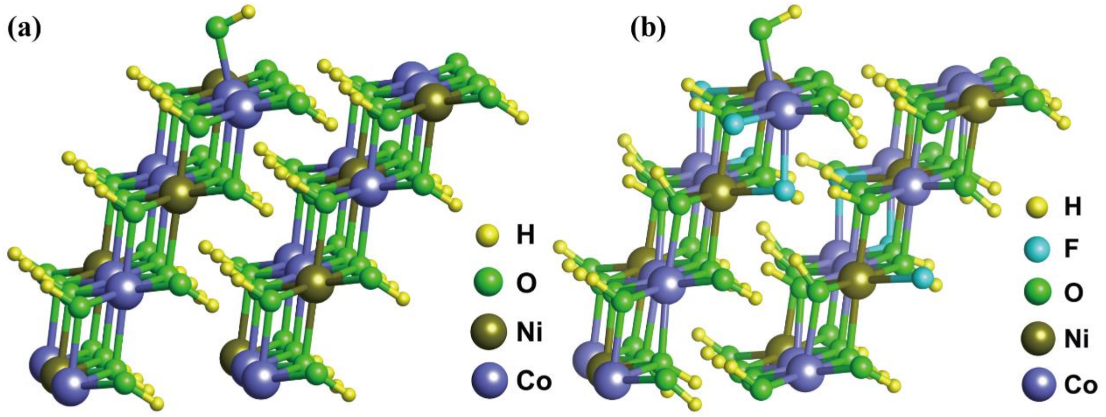

Figure S5. Models of the calculation of OH^- adsorption on NCH (**a**) and FNCH (**b**).

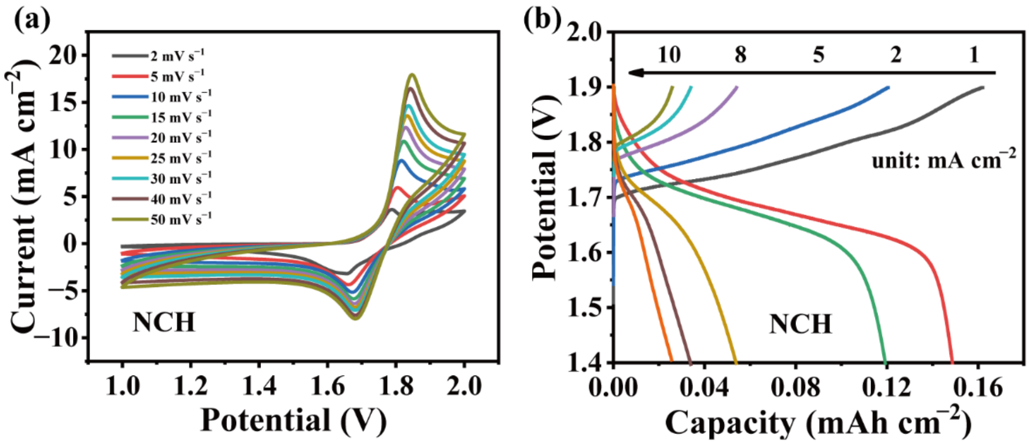

Figure S6. CV curves (**a**) and GCD curves (**b**) of NCH//Zn battery.
